# Beyond the Limits: Clinical Utility of Novel Cardiac Biomarkers

**DOI:** 10.1155/2015/187384

**Published:** 2015-10-04

**Authors:** Radmilo Janković, Danica Marković, Nenad Savić, Vesna Dinić

**Affiliations:** ^1^Center for Anesthesiology and Reanimatology, Clinical Center in Niš, Bulevar Dr. Zorana Djindjića 48, 18000 Niš, Serbia; ^2^Department for Anesthesia and Intensive Care, School of Medicine, University of Niš, Bulevar Dr. Zorana Đinđića 81, 18000 Niš, Serbia

## Abstract

Preoperative assessment of cardiovascular risk is essential when it comes to extensive noncardiac surgery procedures. Therefore, accurate and timely diagnosis of myocyte damage is vital. In modern medical practice it is believed that the so-called “multimarker” approach is the most appropriate and most accurate, but new research points out that there are novel biomarkers which could be used independently. Studies that evaluate miRNA, H-FABP, and MR-PAMP give encouraging results. When it comes to miRNA clinical studies show high statistical significance, especially in the case of acute myocardial infarction (*P* = 0.001). Statistical significance of *P* = 0.007 was found in acute coronary syndrome, when H-FABP was measured. Biochemical marker MR-PAMP showed statistical significance of *P* < 0.0001 in most clinical studies.

## 1. Introduction

Anesthesiologists are daily in contact with patients who are preparing for noncardiac surgery procedures and who are at increased risk to develop cardiovascular complications in perioperative period. The number of these patients is increasing worldwide [1, 2]. Perioperative risk can be estimated based on the severity of existing heart failure, development of recent myocardial infarction, existence of arrhythmias, presence of aortic stenosis, patient's age, type of planned surgery, chronic obstructive pulmonary disease, renal function, previous transient ischemic attack, the general condition of the patient, and so forth [3–6]. Postoperative hypertension, arrhythmia, and heart failure usually occur two days after surgery, while the risk of perioperative myocardial infarction persists during five to six postoperative days [3]. In total, less than 1% of patients develop perioperative myocardial infarction; however about two-thirds of these patients die within 30 postoperative days. Therefore, timely preoperative diagnosis of myocyte damage is vital [1]. Until recently Lee score was used to assess perioperative risk; however European Society of Cardiology (ESC) and European Society of Anaesthesiology (ESA) have proposed a new and comprehensive way to evaluate perioperative cardiac risk [7, 8]. Significant item of this preoperative evaluation and processing of high-risk patients are biomarkers. We have evaluated potential use of miRNA, H-FABP, and MR-PAMP in everyday clinical practice as well as the characteristics which make them stand out as highly promising novel biomarkers.

## 2. Biomarkers in Clinical Practice

Biological marker or biomarker can be objectively measured and it is an indicator of biological processes [8]. From the definition, ideal biomarker has the following characteristics: a high presence in the heart tissue, an absence in other tissues, an absence in the serum of healthy individuals, quick release for the purpose of early diagnosis, a long half-life for the purpose of late diagnosis, cost-effectiveness, and positive evaluation in clinical trials [9–11]. The search for an ideal cardiac biomarker lasts for almost a century (Table 1).

In modern medicine practice there are numerous biomarkers, those that point to an obvious pathology of cardiomyocytes and those that provide a strong evidence of comorbidity such as acute renal failure and pneumonia [12]. The following biomarkers are currently used: aspartate aminotransferase (AST), lactate dehydrogenase (LDH), creatine kinase (CK), hydroxybutyrate dehydrogenase (HBDH), creatine kinase MB isoenzyme (CK-MB), CK-MB mass, myoglobin, carbonic anhydrase, glycogen phosphorylase BB, troponin T (TnT), and troponin I (TnI) [9, 13]. According to the latest recommendations, biomarkers which determine myocardial ischaemia and damage, inflammation, and left ventricular function are used in the perioperative period. The most popular biomarkers in modern practice are cTnI and cTnT due to higher sensitivity and specificity when compared to other biomarkers. Clinical studies have shown that even a small increase in the concentration of cTnT in the perioperative period indicates myocardial damage and worsens postoperative prognosis and outcome. Preoperative determination of BNP and NT-pro-BNP levels indicates the possibility of developing cardiovascular complications after major noncardiovascular surgeries [8, 13–16]. Biomarkers also have a great role in improving the treatment of heart diseases and monitoring of the effectiveness of the therapy [12]. There is the fact that there are no biomarkers which can be interpreted and included in routine work alone but can only indicate the high-risk patients [8].

**Figure 1:**
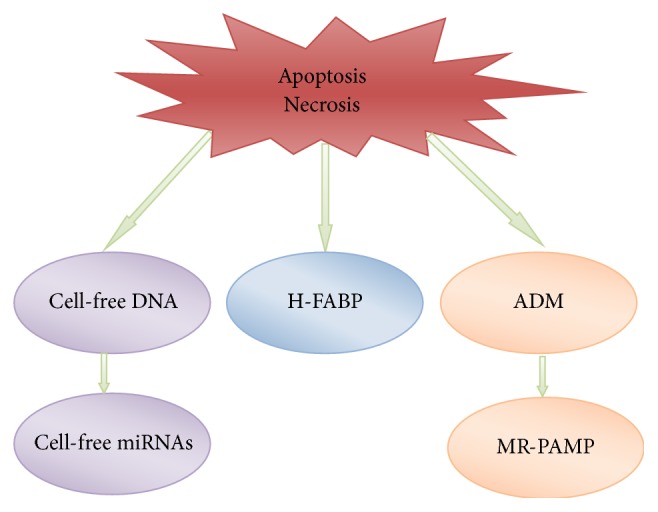
Biomarkers and their precursors in serum after cell apoptosis and necrosis.

It is believed that a multimarker approach is the most appropriate and most accurate and that a new set of biomarkers (currently in the trial phase) is much more accurate and that they could even be used independently [12]. The development and definition of new biomarkers will lead to a faster and more accurate diagnosis of myocardial damage [8, 17]. A large number of studies about new potential biomarkers of myocardial damage such as microRNA (mrRNA), fatty acid-binding protein (FABP), and proadrenomedullin (PAMP) are currently conducted (Figure 1).

## 3. The Significance of miRNA as a Biomarker

MicroRNA (miRNA) represents a recently discovered class of endogenous, small, noncoding RNA molecules which regulate the expression of approximately 30% of genes in the human genome and are highly stable in the circulation [2, 18–22]. Basic and clinical studies indicate a great importance of miRNA in the regulation of cellular differentiation, growth, proliferation, and apoptosis [23–25]. Besides miRNA, which are specific for the majority of tissues, there are also miRNAs with a tissue-specific expression and they could be useful in practice [19, 26, 27].

These molecules can be transported between the cells in the form of miRNA bound to RISC, Argonaute 2, and Nucleophosmin 1 proteins as well as in the form of miRNA packed in vesicles. It is considered that a free miRNA molecule can be found in blood as a consequence of the release of the cell contents in plasma due to necrosis [26].

The role of miRNA was first studied in the field of oncology, where they were considered to be both oncogenes and tumor suppressors [2, 28]. Earlier studies indicated a great expression of miRNA in the heart cells; however their role has not been completely known until the beginning of the research in 2005 [2, 19]. Research based on miR-155 has indicated that it has a key role in the development of immune disorders, tumors, cardiovascular diseases, viral infections, and so forth [29]. It has later been shown that miRNA molecules are essential for the proper development of cardiovascular system [26]. Some of the miRNAs expressed in the heart tissue are miR-21, miR-29a, miR-129, miR-210, miR-211, miR-320, miR-423, and let-7c. In a completely healthy heart tissue miR-1, miR-16, miR-27b, miR-30d, miR-126, miR-133, miR-143, miR-208, and let-7 families are highly expressed [21].

An aberrant presence of miR-21 in the blood vessel wall after balloon damage has been proved by using microarray and Northern blot analysis as well as qRT PCR method [30]. Several studies made miRNA target molecules in the therapy of atherosclerosis, postangioplastic restenosis, transplantation arteriopathy, and cerebral and heart ischaemia [30–32]. Cipollone et al. have examined 41 miRNAs and discovered profound differences in the expression of miR-100, miR-127, miR-145, miR-133a, and miR-133b in symptomatic versus asymptomatic plaques [33].

Myocardial hypertrophy represents a great determinant in predicting the mortality and morbidity in cardiovascular diseases [2]. Significant negative effects on the myocardial hypertrophy were obtained by changing the expression of miR-21 by knockdown method, or antisense-mediated depletion method. It can be concluded that miRNA is involved in the process of myocardial hypertrophy and can represent a future target in the therapy of diseases which include myocardial hypertrophy like hypertension, ischaemic heart disease, valvular heart disease, and endocrine diseases [2]. Studies have shown that miR-1, miR-21, miR-133, miR-195, and miR-208 have the most important role in the development of hypertrophy [25].

Lovren et al. have shown that miRNA-145 is highly expressed in the smooth muscle cells of blood vessels and that its overexpression can lead to the development of atherosclerosis [28]. Other miRNAs expressed in the atherosclerosis are miR-126, miR-145, miR-146a, miR-155, and miR-210 [23, 27]. miRNA-145 molecule controls the differentiation of smooth muscle cells and promotes the formation of lesion; miR-126 sends signals about the need to repair endothelium, while miR-155 indicates the presence of proinflammatory macrophages and atherosclerotic lesions [34, 35]. Research about the influence of miRNA on the development of atherosclerosis is a relatively new field so it is considered that the full clinical potential of this molecule in that area is yet to be demonstrated [26, 34].

Patients with arterial coronary disease have decreased levels of miR-126, miR-145, and miR-155 in circulation. Many studies are currently trying to assess the impact of miR-1, miR-133a, miR-133b, miR-208, miR-499, and miR-499-5p on the development of acute myocardial infarction [21]. It has been proved that miR-208b, miR-499, and miR-320a have been significantly elevated in the patients with acute myocardial infarction; however none of them could be used independently in the diagnostics without cTnT or hsTnT [36]. Wang et al. have reported that elevated levels of cardiac-specific miR-208a in plasma may represent a new biomarker for early detection of myocardial damage [37]. Also circulating miR-1, miR-133a, miR-208b, and miR-499 may be useful biomarkers in acute myocardial infarction, but they are not superior to cTnT [38]. However, according to research circulating miR-499 represents a new early biomarker for the identification of perioperative myocardial infarction in cardiac surgery [39].

Atrial fibrillation is associated with reduced levels of miR-1 in atrial tissue and an overexpression of miR-133 and miR-328 [21, 25]. Cardin et al. have concluded that knockdown of the gene miR-21 in atrium suppresses atrial fibrosis and the onset of atrial fibrillation, and therefore miR-21 is an important signaling molecule for the development of this disorder [40]. It is also considered that miR-1 is responsible for the changes in function of many proteins which take over Ca2+, so it is connected with the onset of ventricular fibrillations [21].

It is considered that miRNAs have a significant impact on the development of hypertension, especially through the influence on the renin-angiotensin-aldosterone system. Some of the significant miRNAs for the hypertension onset are miR-181a, miR-663, miR-132, miR-212, miR-143, and miR-145 [21].

The onset of heart failure is connected to miR-122, miR-210, miR-423, miR-5p, miR-499, and miR-622. Research points out to a high specificity of miR-16, miR-27a, miR-101, and miR-150 in predicting the occurrence of left ventricular failure six months after acute myocardial infarction [21]. Circulating miR-423 and miR-5p are considered potential biomarkers of heart failure because they were in correlation with NT-pro-BNP and ejection fraction of the heart [41, 42].

On the other side, miRNA still has a small diagnostic potential when independent of troponin, and test conduction requires too much time [21]. Also there exist difficulties in practice when it comes to measuring absolute levels of miRNA, the lack of standardized protocols, and variations in test performance depending on the laboratory and heparin in the sample represents a significant inhibitor of reactions based on PCR method. Therefore it is crucial to improve methods and standardize protocols before final introduction of this biomarker in practice [43, 44]. However, it is considered that miRNA could represent an important cardiac biomarker in the near future (Table 2).

## 4. The Significance of H-FABP as a Biomarker

Cytoplasmic FABP represent a family of transport proteins which allow the transport of fatty acids through the membranes. FABP show tissue specificity so there exist liver-type FABP (L-FABP), intestinal-type (I-FABP), brain-type FABP (B-FABP), and heart-type FABP (H-FABP). Expression of H-FABP is also specific for brain tissue while the coexpression of H-FABP and L-FABP is specific for kidney tissue [45]. These biomarkers have found their application in liver rejection and kidney viability assessment, in the diagnosis of inflammatory and ischemic intestinal diseases, in traumatic damage of brain tissue, and in the prevention of muscle damage in those who have intensive physical activity [45].

H-FABP represents a small cytosolic protein that functions as a carrier of long chain fatty acids in cardiomyocytes. It is present in high concentrations in myocardial tissue and is quickly released into the circulation after the damage of the myocardial tissue [46, 47].

When it comes to the patients with chest pain in primary medical care, it is considered that H-FABP is the most specific marker besides a highly sensitive troponin T (hs-cTnT) [48].

This molecule is recommended for the initial diagnosis of myocardial infarction as well as for the evaluation of minimal subclinical myocyte injury [1]. H-FABP has proved to be even more successful than cTnT in research when it comes to the diagnosis of myocardial damage in patients with chronic heart failure [1]. A high diagnostic sensitivity of H-FABP after myocardial damage has been proved, even 93.1% higher than CK-MB and cTnT [25]. Therefore it is considered that detection of positive H-FABP or cTnI indicates the possibility of the onset of myocardial damage [1].

H-FABP represents a highly accurate biomarker for the myocardial tissue damage in acute coronary syndromes and enables detection of minor myocardial tissue damage in the heart failure and unstable angina pectoris [45]. Elevated levels of H-FABP are present in circulation as soon as 2 to 3 hours after the damage and return to normal in 12 to 24 hours after the initial insult [25, 46].

The concentration of myoglobin is much higher in muscle tissue than in myocardial while it is reversed when it comes to H-FABP. Therefore it is considered that H-FABP is more specific biomarker for the assessment of myocardial tissue damage [45]. O'Donoghue et al. have shown that elevated levels of H-FABP are associated with an increased risk of cardiovascular events, onset of heart failure, and death during the first 10 months after the acute coronary syndrome. Moreover, this association is independent of other risk predictors (troponin I, creatinine clearance, patient's history, etc.), age, and sex of the patient [46]. It has been proved that the prognostic significance of H-FABP is highly accurate and it can be successfully interpreted with troponin even in the patients with low and intermediate risk and in the patients with suspected acute coronary syndrome. Studies indicate that H-FABP can be used as a biomarker of myocardial ischemia even in the absence of evident necrosis [49]. H-FABP and TnT are detectable in venous blood of the patients with chronic heart failure with the ongoing myocardial damage. Some of the research show that H-FABP has a higher specificity [47]. H-FABP has shown to be a far more sensitive biomarker when compared to TnT within the first 6 hours after surviving acute heart damage; however, its specificity is reduced after 6 hours [50–53].

It is important to note that not only is H-FABP specific to myocardial tissue, but it is present in skeletal muscle in low concentrations. Most clinicians consider that damage of muscle tissue during the surgery cannot lead to such high levels of H-FABP. However, the damage of kidney tissue during the surgery may result in elevated levels of H-FABP [1]. Also, H-FABP can be elevated in the patients with kidney dysfunction [46].

Further extensive research is necessary in order to evaluate H-FABP as a biomarker of cardiovascular changes and long-term prognosis in the perioperative period (Table 3) [1].

## 5. Significance of Proadrenomedullin as a Biomarker

Adrenomedullin (ADM) is a peptide which has rapid clearance from the circulation and a short half-life (22 minutes) and is not practical to be used as a routine biomarker. Midregional proadrenomedullin (MR-PAMP) is released in higher concentrations than ADM; it is inactive and has a longer half-life; therefore it represents a suitable substitute [12, 54–56].

Molecule PAMP is secreted from rat cardiomyocytes and there exist specific binding places for PAMP in heart tissue. One of its functions is that it is included in the control of coronary vasculatory tone. The level of PAMP is elevated in the patients with essential hypertension and congestive heart failure [14, 55].

Due to its stability and specificity, MR-PAMP is a significant biomarker for the prediction of heart damage since it basically represents a biomarker of endothelial dysfunction [54, 57]. The level of MR-PAMP is elevated in the patients with heart diseases like congenital heart failure, ischaemic heart disease, and atherosclerosis. It also represents a significant predictor of mortality in these individuals [57]. Smith et al. have shown that MR-PAMP, CRP, and NT-pro-BNP are good predictors of the onset of heart failure and that they are completely independent of the other biomarkers and risk factors [58]. The combination of MR-PAMP and Nt-pro-BNP is suggested for the mortality prediction [59, 60].

It has been examined whether a panel of biomarkers (PCT, MR-PAMP, CT-pro-endothelin-1, CT-pro-arginine vasopressin, and MR-pro-ANP), each separately or using the Acute Physiology and Chronic Health Evaluation IV score, has a greater significance in the prediction of intrahospital mortality. It has been shown that the greatest significance in the first 6 to 18 hours after entering the ICU has MR-PAMP, even in the case when its significance has been compared to the Acute Physiology and Chronic Health Evaluation IV score in elective cardiosurgery [61].

Recently published BACH (Biomarkers in Acute Heart Failure) study pointed out that MR-PAMP has a much greater significance than BNP in the mortality prognosis within 90 days in patients with diagnosed acute heart failure [62, 63]. This supports the superiority of MR-PAMP compared to NT-pro-BNP and BNP in the prediction of mortality within 14 days [62–65].

A comparative analysis of 12 biomarkers showed that NT-pro-BNP, GDF-15, MR-PAMP, cystatin C, and MR-pro-ANP are the strongest predictors of cardiovascular complications in patients with stable angina pectoris, alone and in combination [66].

Elevated levels of MR-PAMP indicate the existence of coronary heart disease, heart failure, left ventricular failure, and complications in the septic patients [54]. Studies indicate the possibility of using MR-PAMP as a prognostic biomarker for subclinical and manifest cardiovascular diseases (Table 4) [67].

## 6. Conclusion

Preoperative prediction of cardiovascular risk in patients who are preparing for non-cardiac surgery is essential. New protocols have confirmed that biomarkers have a significant role in that. The “multimarker strategy” is highly present in contemporary clinical practice because there are no independent biomarkers which would timely and accurately indicate the cardiomyocyte damage. The task of future studies is to find the “so-called” ideal biomarker, and studies which evaluate miRNA, H-FABP, and MR-PAMP give encouraging results.

## Figures and Tables

**Table 1:** Brief history of cardiac biomarkers.

Biomarker	Year of first appearance in literature	One of the first significant references when it comes to myocardial injury
H-FABP	1988	[68]
AST	1954	[69]
LDH	1948	[70]
CK	1954	[71]
HBDH	1963	[72]
CK-MB	1962	[73]
CK-MB mass	1986	[74]
Myoglobin	1941	[75]
TnT	1940	[76]
TnI	1987	[77]
miRNA	2009	[37]
PAMP	2002	[78]

**Table 2:** Summary of original articles from the literature proving the significance of miRNA as cardiac biomarker.

Authors	Year	Study type	Number of patients	Statistical significance
Raitoharju et al. [32]	2011	Laboratory study	12 atherosclerotic plaques	*P* < 0.05

Li et al. [31]	2011	Clinical study	104 patients with atherosclerosis obliterans	miR-21, miR-130a, miR-27b, let-7f, and miR-210 significantly increased

Cipollone et al. [33]	2011	Laboratory study	53 atherosclerotic plaques	miR-100, miR-127, miR-145, miR-133a, and miR-133b were more expressed in symptomatic versus asymptomatic plaques

Devaux et al. [36]	2015	Clinical study	224 patients with AMI	miR-208b, miR-499, and miR-320a were significantly higher in patients with AMI

Wang et al. [37]	2010	Clinical study	66 patients	miR-1, miR-133a, miR-499, and miR-208a in AMI *P* < 0.05
			33 AMI, 33 non-AMI with chest pain and distress	

Li et al. [38]	2013	Clinical study	67 patients with AMI	miR-1, miR-133a, miR-208b, and miR-499 in AMI *P* < 0.001

Yao et al. [39]	2014	Clinical study	30 on-pump patients after coronary artery bypass surgery	miR-499, *P* = 0.001; miR-133a, *P* = 0.006; miR-133b, *P* = 0.05

**Table 3:** Summary of original articles from the literature proving the significance of H-FABP as cardiac biomarker.

Authors	Year	Study type	Number of patients	Statistical significance
Sari et al. [1]	2015	Clinical study	67 patients, 40 with diabetes	*P* = 0.01
O'Donoghue et al. [46]	2006	Clinical study	2287 patients with acute coronary syndrome	Elevated in 332 patients (14.5%)
Niizeki et al. [47]	2007	Clinical study	126 patients with chronic heart failure	*P* < 0.001
Willemsen et al. [48]	2015	Clinical study	202 patients with acute coronary syndrome	AUC 0.79 versus 0.80
Viswanathan et al. [49]	2010	Clinical study	1080 patients with suspected acute coronary syndrome	*P* = 0.007
Ruzgar et al. [50]	2006	Clinical study	40 patients with suspected acute coronary syndrome	*P* = 0.014
Mad et al. [51]	2007	Clinical study	22 patients with AMI	*P* < 0.05
			20 with unstable angina and 15 with noncardiac chest pain	
Liao et al. [52]	2009	Clinical study	74 patients with AMI	*P* < 0.05
Haltern et al. [53]	2010	Clinical study	97 with acute ischaemic chest pain	*P* < 0.05

**Table 4:** Summary of original articles from the literature proving the significance of MR-PAMP as cardiac biomarker.

Authors	Year	Study type	Number of patients	Statistical significance
Eggers et al. [57]	2013	Cohort study	1797 patients over 70 years of age	*P* < 0.001
Smith et al. [58]	2010	Cohort study	5187 individuals	Heart failure, *P* < 0.03; atrial fibrillation, *P* < 0.001
Von Haehling et al. [59]	2007	Clinical study	525 patients with chronic heart failure	*P* < 0.0001
Von Haehling et al. [60]	2010	Clinical study	501 patients with chronic heart failure	*P* < 0.0001
